# Mediating effect assessment of ifosfamide on limb salvage rate in osteosarcoma: A study from a single center in China

**DOI:** 10.3389/fonc.2022.1046199

**Published:** 2022-11-03

**Authors:** Yan Li, Yiwei Fu, Zhaohui Zhang, Zhuo Wang, Junqiang Yin, Jingnan Shen

**Affiliations:** ^1^ Department of Musculoskeletal Oncology, The First Affiliated Hospital of Sun Yat-sen University, Guangzhou, China; ^2^ Department of Radiology, The First Affiliated Hospital of Sun Yat-sen University, Guangzhou, China; ^3^ Department of Pathology, The First Affiliated Hospital of Sun Yat-sen University, Guangzhou, China

**Keywords:** ifosfamide, osteosarcoma, limb salvage, amputation, neoadjuvant chemotherapy

## Abstract

Osteosarcoma is one of the most prevalent primary bone malignancies in children and adolescents. Surgery and chemotherapy are the standard treatment methods of osteosarcoma. Methotrexate, adriamycin, and cisplatin, and methotrexate, adriamycin, cisplatin, and ifosfamide regimens are both first-line neoadjuvant chemotherapy regimens for osteosarcoma. Moreover, the use of ifosfamide is highly controversial. Most studies of ifosfamide focused on the overall survival rate and event-free survival rate; few studies concentrated on surgical options. We conducted this retrospective study to compare the baseline characteristic of amputation and limb salvage osteosarcoma patients. Furthermore, we analyzed the direct and indirect roles in surgical decision-making and found that ifosfamide may play a partial mediating role in the surgery option choice by mediating tumor mass volume change, tumor response, and the shortest distance from the center of main blood vessels to the margin of the tumor lesion.

## Introduction

Osteosarcoma is the most common primary malignant bone tumor with a particular risk in children and adolescents. Since the concept of neoadjuvant chemotherapy was proposed, great progress has been made in the diagnosis and treatment of osteosarcoma. However, there has been little progress in neoadjuvant chemotherapy regimens in recent decades, and most current studies on neoadjuvant chemotherapy mainly focus on the impact of the overall survival (OS) rate and the event-free survival (EFS) rate (1). The addition of neoadjuvant chemotherapy to complement surgical resection increased the 5-year OS rate to 60%–70% for adolescents with localized disease (2). Methotrexate, adriamycin, and cisplatin (MAP) are commonly used as a base regimen, whereas the methotrexate, adriamycin, cisplatin, and ifosfamide (MAPI) regimen is accepted as the first line and is widely used in patients as well (3). The influence of different neoadjuvant chemotherapy regimens on the choice of surgery has been poorly clarified.

Compared with amputation surgery, limb salvage has become a preferred option for osteosarcoma with the advancement of surgical technology and the neoadjuvant chemotherapy regimen and is reported to have a better 5-year OS rate (4, 5). Limb salvage rates for osteosarcoma continue to increase owing to constantly improved curation including neoadjuvant chemotherapy, ablation techniques, bone transport techniques, radiotherapy regimens (6), and computer navigation techniques (7–10). There are many factors that may influence the choice of surgical methods between limb salvage and amputation (11): apart from the tumor mass volume (TMV) and tumor stage, the multidisciplinary approach involving imaging, meticulous surgical procedures may have a great influence on the surgery choice for pediatric osteosarcoma (12, 13). Although the limb salvage rate of osteosarcoma has been improved up toward 80%–90%, there is still a considerable amputation incidence at approximately 15% in children (5) and 15%–20% in the elderly population (14), which may decrease their quality of life. Neoadjuvant chemotherapy can improve the probability of limb salvage surgery (15, 16), but, up until now, there is little research on the relationship between the neoadjuvant chemotherapy regimen and surgical options.

Our study stands from the surgeons’ points of view and takes the operative options of limb salvage or amputation as the object of comparison, by comparing the MAP and MAPI regimens, to explore whether ifosfamide may contribute to improve the limb salvage rate of osteosarcoma.

## Patients and methods

### Study population

Patients with a clinic diagnosis of osteosarcoma at The First Affiliated Hospital of Sun Yat-sen University from January 2008 to February 2021 were enrolled in this retrospective study.

The inclusion criteria were as follows: (1) patients who were first diagnosed or suspected as osteosarcoma in our hospital histologically (by needle biopsy); (2) patients who received MAP or MAPI neoadjuvant chemotherapy treatment; and (3) patients with relatively complete clinical data, including age at diagnosis, sex, the site of the primary tumor, the existence of pathological fracture at diagnosis, the Enneking stage, histology, and the tumor volume before and after neoadjuvant chemotherapy.

**Figure 1:**
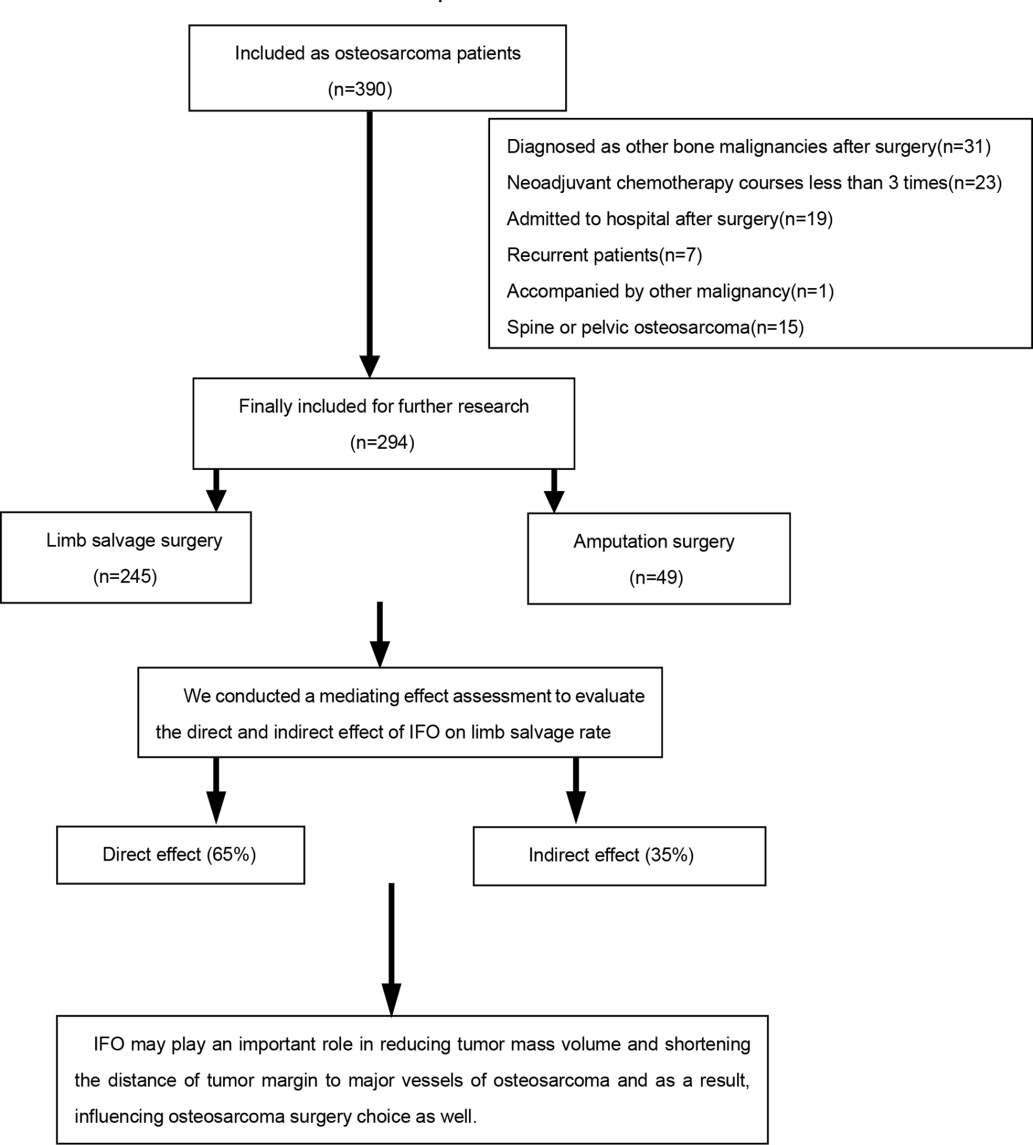
Patient Screening Flowchart.

The exclusion criteria were as follows: (1) patients with incomplete data (data missing rate>20% according to the specified entry); (2) patients who were not really osteosarcoma patients in a postoperative pathological examination; (3) patients with a non-standardized chemotherapy regimen, which are not consistent with our center (17) or who have not received neoadjuvant chemotherapy at all; (4) recurrent osteosarcoma patients; and (5) patients accompanied with other malignancy (**Figure 1**).

This study was approved by the Medical Ethics Committee of The First Affiliated Hospital of Sun Yat-sen University (No [2021].304), and the requirement for informed consent was waived because of the retrospective nature of the study.

### Chemotherapy and surgeries

The conventional chemotherapy regimen is mainly a methotrexate, adriamycin, cisplatin, and ifosfamide (MAPI) regimen or a methotrexate, adriamycin, and cisplatin (MAP) regimen in our center (17), which included at least three courses of neoadjuvant chemotherapy; each course consisted of the intravenous administration of methotrexate 8–12 g/m^2^ over 4 h (maximum dose 20 g), cisplatin 100–120 mg/m^2^ followed by adriamycin 50–70 mg/m^2^. The MAPI group also received at least one course of Ifosfamide (IFO) (4 h infusion of 1.5–2.0g/m^2^ per day for 5 days). Ifosfamide usage was randomized.

In general, the principles that will guide the decision are as follows: limb salvage should offer the same chance of cure as amputation; the reconstruction used should be acceptable to both the child and the parents; the risk of short- and long-term complications should be acceptable; and the functional and cosmetic outcome should be equal to, or better than, that from amputation (3).

### Collection and clinical information

We collected clinical data, including age at diagnosis, sex, the location of the primary tumor, the pathological stage, the existence of pathological facture at diagnosis, histology, the TMV and TMV shrinkage rate before and after neoadjuvant chemotherapy, and survival information. The diagnosis, histologic subtype, and histologic response were reviewed by pathologists who are experts in sarcoma histology.

**Figure 2:**
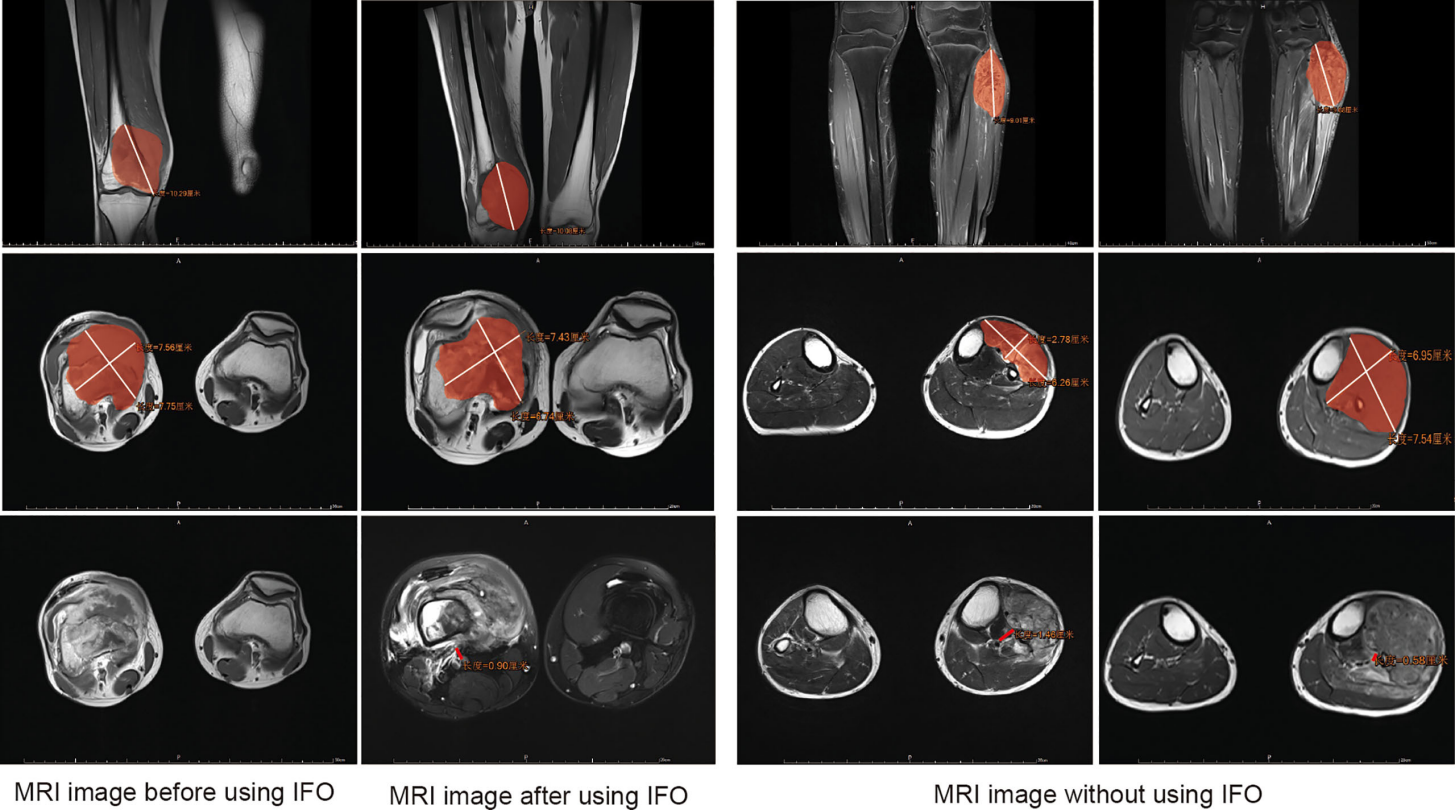
Assessment of the tumor mass volume (TMV) and the shortest distance from the center of main blood vessels to the margin of the tumor lesion (NBT) in MRI. Area covered in red represents the tumor area.

Demographic information was obtained from the electronic medical record system of The First Affiliated Hospital of Sun Yat-sen University. We defined the TMV as the tumor mass volume (soft tissue tumor volume included but reactive area not included). In this article, we define main blood vessels as blood vessels that have been anatomically named, including the main branches of the axillary, brachial, radial, ulnar, femoral, popliteal, and posterior tibial artery and vein (13). NBT is the shortest distance from the center of main blood vessels to the margin of the tumor lesion (**Figure 2**). The TMV and its vascular relationship are measured from MRI, and for the main vessels that are away from the reactive area, we classified them into the >5 mm subgroup (Type 1) and ≤5 mm subgroup (Type 2). Vessels within the tumor-reactive area but not embedded in the tumor are defined as “attach” (Type 3); blood vessels surrounded by tumors are defined as “surrounded” (Type 4) (12, 13). The presence of pathological fracture is obtained from the X-ray image. The TMV was calculated using the following formula: TMV = π/6 (longitudinal maximum diameter × anteroposterior maximum diameter^2^) (18, 19). TMV change (%) = (prechemotherapy TMV − postchemotherapy TMV)/prechemotherapy TMV * 100%. We have classified the tumor volume change into three stages: 1) TMV decrease >10%; 2) TMV change between ±10% (with ±10% included); and 3) TMV increase >10% according to the volume we described before.

In order to find out which factors influence surgery options, in this retrospective study, we defined patients receiving surgery as our outcome index and divided surgical options into limb salvage and amputation surgery depending on a patient’s specific situation.

### Statistical analysis

Categorical variables are expressed as numbers (percentages); Fisher’s exact test and chi-square analysis were used to analyze categorical data. Means were compared using *t*-test assuming unequal variances. The Kaplan–Meier and log-rank tests were applied to draw and evaluate the significance of OS curves.

We took surgery (limb salvage or amputation) as our outcome indexes and performed a statistical analysis of the clinical baseline levels of patients in the amputation group and the limb salvage group. Univariate and multivariate logistic analyses were performed to identify the probable predictor factors of the surgery option. Variables that were found to be significant in univariate analysis were included in multivariate logistic analysis. A two-sided *p*-value < 0.05 was considered statistically significant.

Multiple imputation was used to fill in the missing values of covariates for the study sample to avoid a decrease in statistical power and to minimize selection bias (20). We performed mediating-effect analysis to find out if IFO had a direct or indirect effect on surgery options.

Statistical analyses and graphics were performed with R 4.1.2 (The R Foundation for Statistical Computing, Vienna, Austria) with statistical analysis packages.

## Results

A total of 294 patients were included in this study (**Table 1**), among which 245 patients underwent limb salvage surgery, whereas 49 patients underwent amputation. The limb salvage rate was 83.3%, and the amputation rate was 16.7%.

**Table 1:** Clinical characteristics of patients receiving limb salvage versus amputation for osteosarcoma.

		Type of surgery		
Characteristics		Limb salvage	Amputation	*p*
Total		245 (83.3)	49 (16.7)	
Sex	Women	101 (41.2)	18 (36.7)	0.671
	Men	144 (58.8)	31 (63.3)	
Age	≤20	196 (80.0)	36 (73.5)	0.425
	21–40	43 (17.6)	11 (22.4)	
	41-60	6 (2.4)	2 (4.1)	
	>60	0 (0.0)	0 (0.0)	
Tumor site	femur	146 (59.6)	32 (65.3)	0.967
	fibula	5 (2.0)	1 (2.0)	
	humerus	18 (7.3)	74 (8.2)	
	metatarsal	1 (0.4)	0 (0.0)	
	radius	2 (0.8)	0 (0.0)	
	tibia	71 (29.0)	12 (24.5)	
	ulna	2 (0.8)	0 (0.0)	
Pathological fracture	no	230 (93.9)	48 (98.0)	0.487
	yes	15 (6.1)	1 (2.0)	
NBT	Type 1	114 (46.5)	19 (38.8)	0.106
before neoadjuvant chemotherapy	Type 2	114 (46.5)	25 (51.0)	
	Type 3	16 (6.5)	3 (6.1)	
	Type 4	1 (0.4)	2 (4.1)	
NBT after neoadjuvant chemotherapy	Type 1	27 (11.0)	1 (2.0)	<0.001
	Type 2	105 (42.9)	6 (12.2)	
	Type 3	107 (43.7)	32 (65.3)	
	Type 4	6 (2.4)	10 (20.4)	
Enneking stage	IIB	215 (87.8)	41 (83.7)	0.483
	III	30 (12.2)	8 (16.3)	
TMV before neoadjuvant chemotherapy	≤200 cm^3^	34 (13.9)	9 (18.4)	0.55
	>200 cm^3^	211 (86.1)	40 (81.6)	
TMV after neoadjuvant chemotherapy	≤200cm^3^	69 (28.2)	13 (26.5)	<0.95
	>200 cm^3^	176 (71.8)	36 (71.5)	
TMV change	Decrease >10%	138 (56.3)	15 (30.6)	0.002
	± 10%	28 (11.4)	12 (24.5)	
	Increase >10%	79 (32.2)	22 (44.9)	
Tumor response	PD	72 (29.4)	28 (57.1)	0.001
	PR	106 (43.3)	12 (24.5)	
	SD	67 (27.3)	9 (18.4)	
Chemotherapy regimen	MAP	26 (10.6)	20 (40.8)	<0.001
	MAPI	219 (89.4)	29 (59.2)	

There were no statistical differences in sex, age, tumor site, pathological fracture, and the Enneking stage (*p* > 0.05).

In our baseline data, tumor response [based on the Response Evaluation Criteria In Solid Tumors (RECIST) score] and proximity to main vessels after neoadjuvant chemotherapy were likely to affect the limb salvage surgery choice (*p*<0.05), which were consistent with our cognition and previous research data.

Regarding the changes in the tumor volume of patients before and after neoadjuvant chemotherapy, we divided the TMV changes of patients into three grades: tumor volume increases by 10%, tumor volume decreases by more than 10%, and tumor volume remains stable (between -10% and +10%) (**Table 1**). Results of the statistical analysis found that patients with a tumor volume shrinkage decrease of more than 10% and whose tumor volume remained stable were more likely to receive limb salvage surgery, and patients with a tumor volume increase of more than 10% had higher amputation rates (*p*=0.002).

We investigated the relationship between the tumors and main blood vessels of patients receiving limb salvage surgery and amputation surgery. There were no differences before neoadjuvant chemotherapy, but there were main differences after neoadjuvant chemotherapy (*p*<0.001). These results were consistent with the clinical research previously carried out by our team (13) (**Table 1**).

We observed that tumor volume change was associated with the limb salvage surgical option (**Table 1**, *p*=0.002), and the most relevant factor for tumor volume reduction may be the use of a neoadjuvant chemotherapy regimen; therefore, we wanted to further investigate the use of a neoadjuvant chemotherapy regimen and its relationship with tumor volume change in patients. In our retrospective analysis, according to the The Response Evaluation Criteria In Solid Tumors (RECIST) guideline (version 1.1) (21), we used the TMV when calculating the effect of neoadjuvant chemotherapy drugs on tumor volume change.

**Figure 3:**
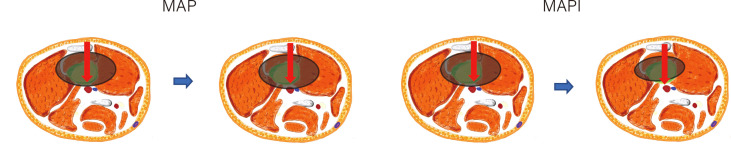
Schematic diagram of effects of IFO on the TMV and NBT after neoadjuvant chemotherapy.

**Figure 4:**
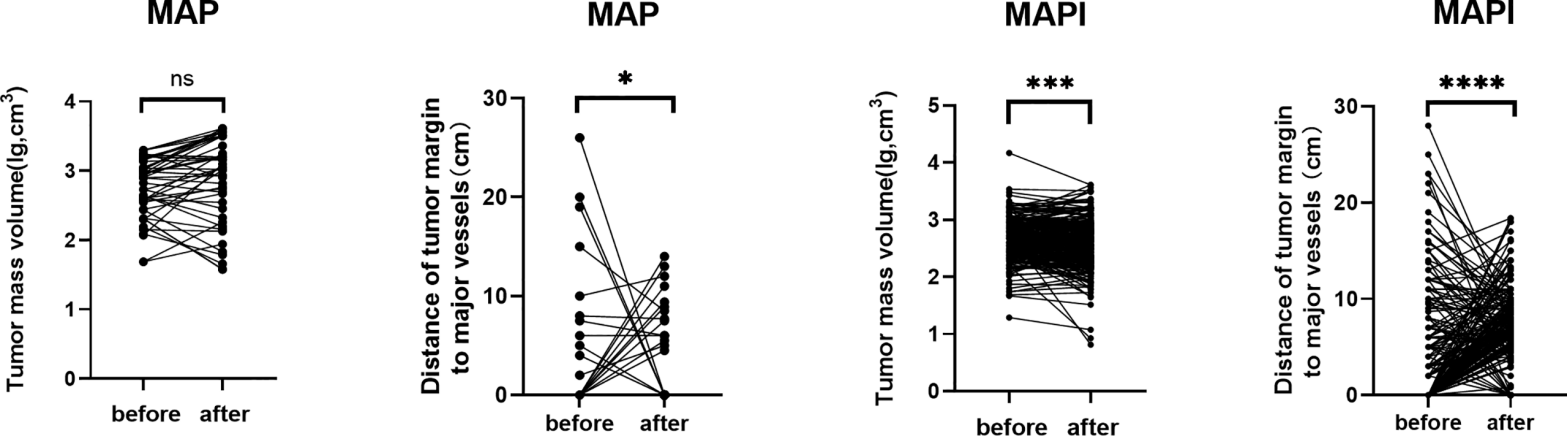
Statistical analysis of effects of IFO on the TMV and NBT after neoadjuvant chemotherapy. *p<0.05; ***p<0.001; ****p<0.0001; ns, no significance.

The assessment of the TMV and NBT in MRI is shown in **Figure 2**, and we also clarified how we calculated the longitude of tumor mass (white line) and the tumor mass area (covered in red). In order to clarify the relationship of TMV change and NBT change, we drew a schematic diagram as seen in **Figure 3**. When IFO was included in the neoadjuvant chemotherapy regimen, the TMV and NBT were significantly reduced after neoadjuvant chemotherapy (*p*<0.001). A statistical analysis of the effects of IFO on the TMV and NBT before and after neoadjuvant chemotherapy is shown in **Figure 4**. A significant statistical difference was shown in the MAPI group. More accurate data are available in **Supplementary Tables 1**–**4**.

**Figure 5:**
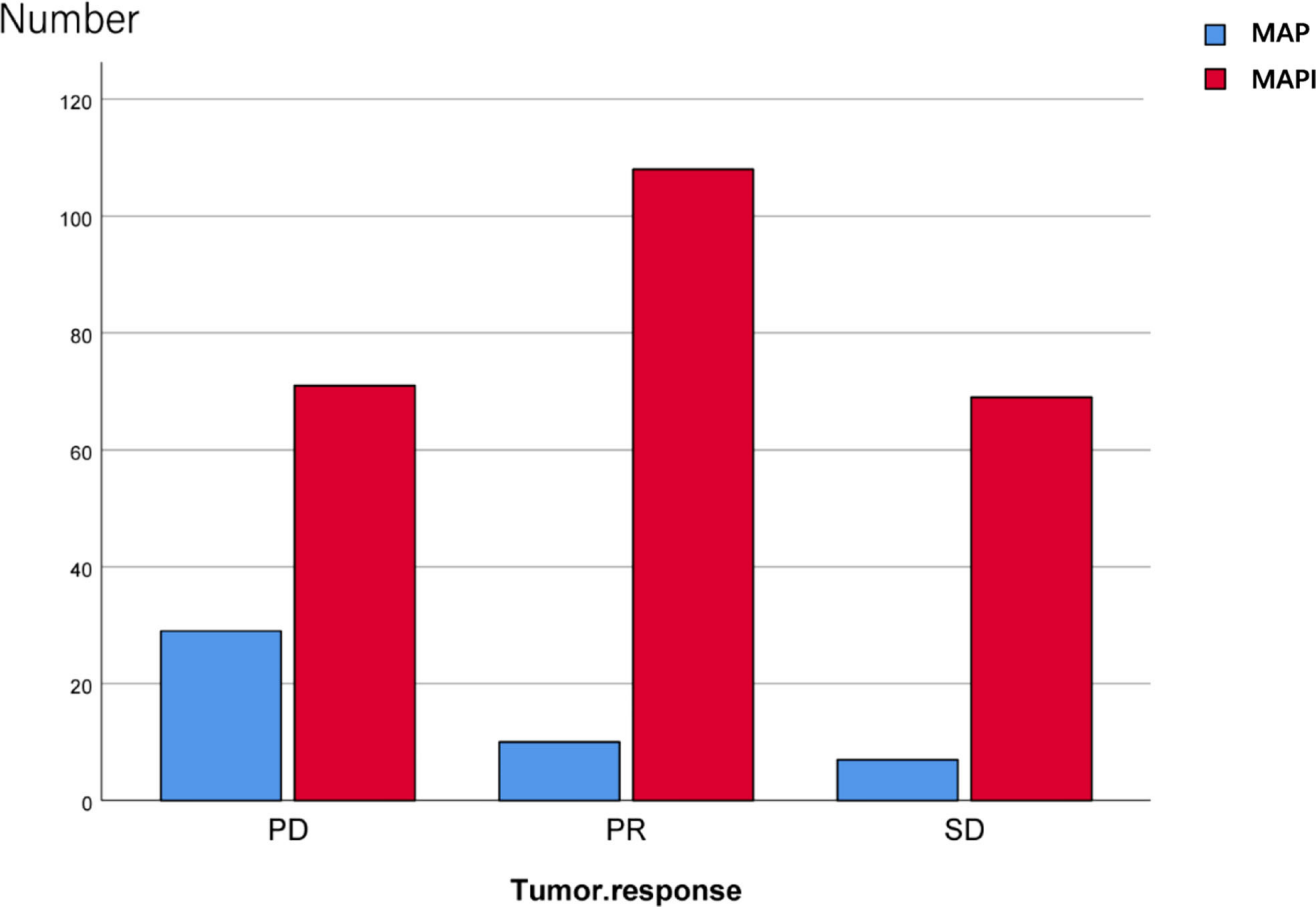
Effects of IFO on the tumor response after neoadjuvant chemotherapy.

We also conducted an analysis of the IFO impact on the tumor response; there exist significant statistical differences as well (*p*<0.001) (**Figure 5**; **Table 2**).

**Table 2:** Statistical analysis of effects of IFO on tumor response before and after neoadjuvant chemotherapy.

Tumor Response	MAP	MAPI	Total	*p*
PD	29	71	100	<0.001
SD	7	69	76	
PR	10	108	118	
Total	46	248	294	

**Figure 6:**
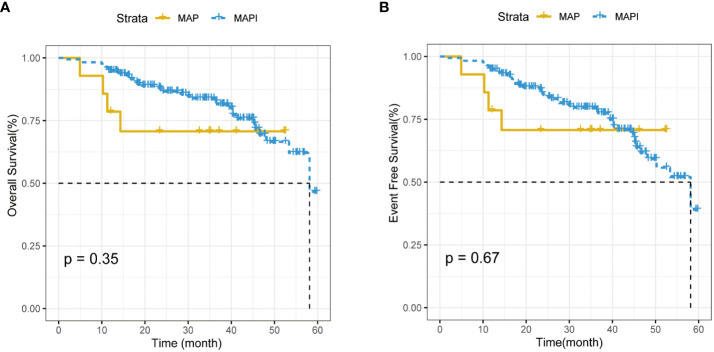
**(A)**. The impact of IFO usage times on the overall survival (OS) rate. **(B)**. The impact of IFO and IFO usage times on event-free survival (EFS) rate.

After carrying Kaplan–Meier survival analysis, the 5-year OS rate and the EFS rate of patients using a MAP or MAPI regimen manifested no difference (*p*>0.05) (**Figures 6A**, **B**).

As our results manifested that IFO may influence the TMV before and after neoadjuvant chemotherapy as well as NBT, we suspected that IFO may also be an influencing factor for the limb salvage surgery choice of osteosarcoma, and, in order to explore the factors that may determine whether patients accept limb salvage surgery or amputation surgery, we performed univariate and multivariate logistic regression analyses on the statistical results (**Table 3**). The results are described below.

**Table 3:** Univariate and multivariate logistic regression model of predictors for receiving amputation.

Characteristic		Univariate analysis			Multivariate analysis		
		OR	CI	*p*-value	OR	CI	*p*-value
Sex	Women	1.00^*^					
	Men	1.21	0.65–2.31	0.56			
Age	≤20	1.00^*^					
	21-40	1.39	0.63–2.88	0.39			
	41-60	1.81	0.26-8.24	0.48			
Tumor site	femur	1.00^*^					
	fibula	0.91	0.05-5.92	0.93			
	humerus	1.01	0.28-2.94	0.98			
	metatarsal	0	NA	0.99			
	radius	0	NA	0.99			
	tibia	0.77	0.36-1.55	0.48			
	ulna	0	NA	0.99			
Pathological fracture	no	1.00^*^					
	yes	0.32	0.10-1.64	0.28			
				0.23			
NBT	Type 1	1.00^*^					
before	Type 2	1.31	0.68-2.52	0.40			
	Type 3	1.12	0.29-4.23	0.86			
chemotherapy	Type 4	12.0	1.03-138.9	0.04			
				<0.001			<0.001
NBT	Type 1	1.00^*^			1.00^*^		
after	Type 2	1.54	0.17-13.36	0.69	1.18	0.13-10.58	0.88
neoadjuvant	Type 3	8.07	1.05-61.76	0.04	6.52	0.83-51.26	0.74
chemotherapy	Type 4	45.0	4.81-421.80	0.01	21.67	2.15-217.75	0.009
Enneking stage	IIB	1.00^*^					
	III	1.39	0.56-3.14	0.44			
TMV before neoadjuvant chemotherapy	≤200 cm^3^	1^*^					
	>200 cm^3^	0.71	0.31-1.68	0.41			
TMV after neoadjuvant chemotherapy	≤200 cm^3^	1^*^					
	>200 cm^3^	1.08	0.54-2.17	0.81			
Tumor response				0.001			0.025
	SD	1.00^*^			1.00^*^		
	PD	2.89	1.27-6.58	0.01	13.89	1.97-97.67	0.008
	PR	0.71	0.33-2.10	0.71	1.06	0.26-4.38	0.92
TMV change				0.003			0.031
	Increase>10%	1.00^*^			1.00^*^		
	± 10%	1.53	0.64-3.52	0.306	14.26	1.97-97.67	0.009
	Decrease >10%	0.39	0.19-0.79	0.01	1.06	0.26-4.38	0.92
Chemotherapy regimen	MAP	1.00^*^			1.00^*^		
	MAPI	0.16	0.08-0.34	<0.001	0.34	0.14-0.81	0.014

OR, odds ratio; CI, confidence interval. ^*^Reference value. NA, Not Available.

In univariate regression analysis, compared with main vessels surrounded by tumors, we found that the long NBT distance is a harmful factor for patients’ surgical choice (Surround: OR=45.0, Attach: OR=8.07, ≤5 mm: OR=1.54, >5 mm: reference, *p*<0.001). Tumor volume change was also an important factor for the amputation surgery option (*p*<0.05) (**Table 3**).

A multivariable analysis was constructed to adjust for these factors that were found to be significant in univariate analyses: compared with the tumor being encapsulated by well-known blood vessels, NBT more than or equal to 5 mm was a protective factor to avoid amputation (*p*<0.05) (**Table 3**).

Apart from all these, we have also found that IFO played as a protective factor in not only univariate logistic regression but also multivariate logistic regression (OR=0.16, 95%CI=0.08-0.34, *p*<0.001; OR=0.34, 95%CI=0.14-0.81, *p*=0.014), which indicated that IFO may be an independent protective factor for osteosarcoma patients to avoid amputation surgery (**Table 3**).

In univariate and multivariate regression analyses, IFO acted as a protective factor. In our baseline data, IFO had an effect on surgery options and, because we had come to the fact that IFO may influence the TMV shrinkage rate and NBT as well as the tumor response. In multiple regression analysis, we noticed that the TMV shrinkage rate, NBT, and tumor response may influence surgery options as well, so we carried out a mediating-effect analysis of these factors.

Our results manifested that the direct effect of the independent variable IFO on the dependent variable “surgery” was -1.3050, accounting for 65% of the total effect; the indirect effect of the independent variable on the dependent variable through the mediator variable 1 (TMV change) was 0.0469, accounting for 23.00% of the total effect. The indirect effect of mediator variable 2 (tumor response) on the dependent variable is -0.1991, accounting for 9.90% of the total effect; the indirect effect of mediator variable 3 (NBT) on the dependent variable is -0.5492, accounting for 27.00% of the total effect. The 95% confidence interval of mediator variable 1 (TMV change) contains 0; that is, the chain-mediating effect of mediator 1 independent variable and the dependent variable is not significant (**Figure 5**; **Table 3**).

## Discussion

Osteosarcoma is a kind of mesenchymal originated malignant tumor that affects mostly children, adolescents, and young adults (22). The treatment of osteosarcoma has always been a thorny problem. Before the 1970s, the curation of osteosarcoma mainly involved limb resection. The introduction of chemotherapy had greatly improved the clinical outcome of patients with localized osteosarcoma (11). Since the mid-1970s, neoadjuvant chemotherapy and chemotherapy, radiotherapy, and wide resection surgical strategies had not only elevated the 5-year OS rate of OS to 70%–80% (15) but also improved the patient’s limb salvage rate as well as limb function and living quality (5, 23, 24). However, in recent decades, the treatment of patients with osteosarcoma has been at a plateau. Although neoadjuvant chemotherapy regimen has progressed, MAP and MAPI regimens are still the preferred recommendation in the guidelines. Further improving the OS rate and limb salvage rate of patients with osteosarcoma is still one of the directions of the efforts of bone oncologists. The argumentation of the osteosarcoma chemotherapy regimen always exists, among which what matters most is the introduction of the use of IFO: Hartwich et al. initially used IFO in neoadjuvant chemotherapy with a 25% overall response (25–29) and then had been widely used. However, in the treatment of IFO in osteosarcoma, whether the usage of IFO addition may improve OS and EFO remains uncontroversial (**Figure 6A**, **B**); meanwhile, the administration of IFO had been proven to be associated with a number of acute toxic effects, for example, neutropenia, thrombocytopenia, nausea, vomiting, alopecia, and hypersensitivity reactions. Ifosfamide has also been responsible for a series of more specific, potentially life-threatening toxicities: hemorrhagic cystitis, nephropathy, encephalopathy, and cardiac toxicity (29–32). Zhang T et al. found that the MAPIE regimen may perform well in OS and EFS, and it seemed to have the optimal efficiency in relapse and the lung-metastasis osteosarcoma (33). To sum up, most of the current studies on osteosarcoma focus on how to improve the long-term survival of patients with osteosarcoma, but, up until now, there is little research on the relationship between the neoadjuvant chemotherapy regimen and its influence on the surgical options of limb salvage or amputation.

We tried to evaluate various factors of the patients’ baseline information before surgery standing from a surgeon’s position with the aim to clarify the related factors of surgery options and to improve the limb salvage rate and the quality of life in osteosarcoma patients. In our research, we had found that IFO as a neoadjuvant chemotherapy regimen may better shrink the TMV, shorten the distance of the tumor from main blood vessels, affect tumor response, and thus be a protective factor in improving the limb salvage rate of osteosarcoma.

Our baseline data had shown that the proximity to the main vessels was a factor that affected the surgical approach of osteosarcoma. Patients with tumors that were away from tumor margins were more likely to accept the limb salvage surgery, which was consistent with previous research (12). Other factors did not play significant roles in surgery options, such as pathological fracture, the same with the conclusion of the Consecutive Cooperative Osteosarcoma Study Group (COSS) research (34, 35)(**Table 1**).

In our baseline data, we had found that TMV changes and the NBT may be factors that made contributions to surgery options. Preoperative neoadjuvant chemotherapy was a factor that affected TMV reduction and thus shortened the NBT distance. When evaluating the effect of the neoadjuvant chemotherapy regimen, we measured the TMV according to the The Response Evaluation Criteria In Solid Tumors (RECIST) guideline (version 1.1) and found that Ifosfamide (IFO) significantly reduced the TMV after neoadjuvant chemotherapy, made NBT closer, and influenced tumor response(*p*<0.05) (**Figures 2**–**5**).

To further explore whether Ifosfamide (IFO) is an independent factor affecting the surgical approach of osteosarcoma, we carried out a univariate analysis of our baseline data. The results showed that, consistent with the baseline data analysis in **Table 1**, NBT is a factor that may influence the surgery decision-making, and the closer it is from the tumor margin, the more likely that patients receive amputation surgery (*p*<0.05). Apart from these factors, we had also found that a neoadjuvant chemotherapy regimen with or without Ifosfamide (IFO) also made contributions to the decision-making of a surgical approach. As mentioned in **Table 4**, a neoadjuvant chemotherapy regimen with Ifosfamide (IFO) plays as a protective factor in our research (OR=0.16,95%CI=0.08-0.34, *p*=0.002), thus leading to patients who are less likely to receive amputation surgery. Meanwhile, in the multivariate analysis of factors leading to the decision-making of the surgery choice, we had found that apart from NBT and TMV after neoadjuvant chemotherapy, IFO usage also plays a protective role in surgery options (OR=0.34, 95%CI=0.14-0.81, *p*=0.014). To this extent, we may have confidence coming to a conclusion that IFO usage may be an independent protective factor in the surgical options of osteosarcoma.

**Table 4:** Direct and indirect effect of IFO on surgery.

Direct effect of IFO on surgery					
Effect	SE	Z	*p*	LLCI	ULCI
-1.3050	0.4052	-3.2208	0.0013	-2.0992	-0.5109
Indirect effect of IFO effect on surgery					
	Effect	BootSE	BootLLCI	BootULCI	
Total	-0.7015	0.2779	-1.3381	-0.2548	
TMV change	0.0469	0.1352	-0.1919	0.3518	
Tumor response	-0.1991	0.1906	-0.6534	0.0855	
NBT	-0.5492	0.2282	-1.0903	-0.1846	

In order to make out whether IFO influences surgery option directly or indirectly, we carried out the mediating effect analysis of IFO and surgery options, and the mediating variables are TMV change, tumor response, and NBT after neoadjuvant chemotherapy. Our results showed that IFO may influence the surgery choice 65% directly and 35% indirectly by influencing tumor volume change, NBT, and tumor response (*p<*0.05).

Unlike other studies of chemotherapy regimens related to osteosarcoma focusing only on the overall survival and EFS, our research concentrated on the influence of chemotherapy regimens on surgery options or the limb salvage rate. Our result was able to reveal that although IFO had side effects and may not improve overall survival, it may make a contribution to TMV shrinkage and, as a result, improve the limb salvage rate of osteosarcoma, providing evidence for clinical usage in the neoadjuvant chemotherapy treatment of osteosarcoma.

The advantage of our study was based on the surgeon’s point of view. For the first time, the choice of surgery was used as a way of evaluating neoadjuvant chemotherapy outcomes. It was found that the limb salvage rate of patients with the use of IFO significantly improved in comparison with patients without the use of IFO, which may have a relationship with the TMV shrinkage rate. We had put forward new ideas of IFO usage, which may provide clinicians with evidence to weigh the pros and cons when making surgery choices.

There were some limitations in our research: as a clinical retrospective study, the evidence level was limited in comparison with randomized controlled trials or other kinds of clinical trials. Due to the limited sample size and unbalanced cases (few amputees and few patients without the use of IFO), and missing data in the image information, our research may have limitation or some potential bias but can also reflect the real-world scene to some extent.

**Figure 7:**
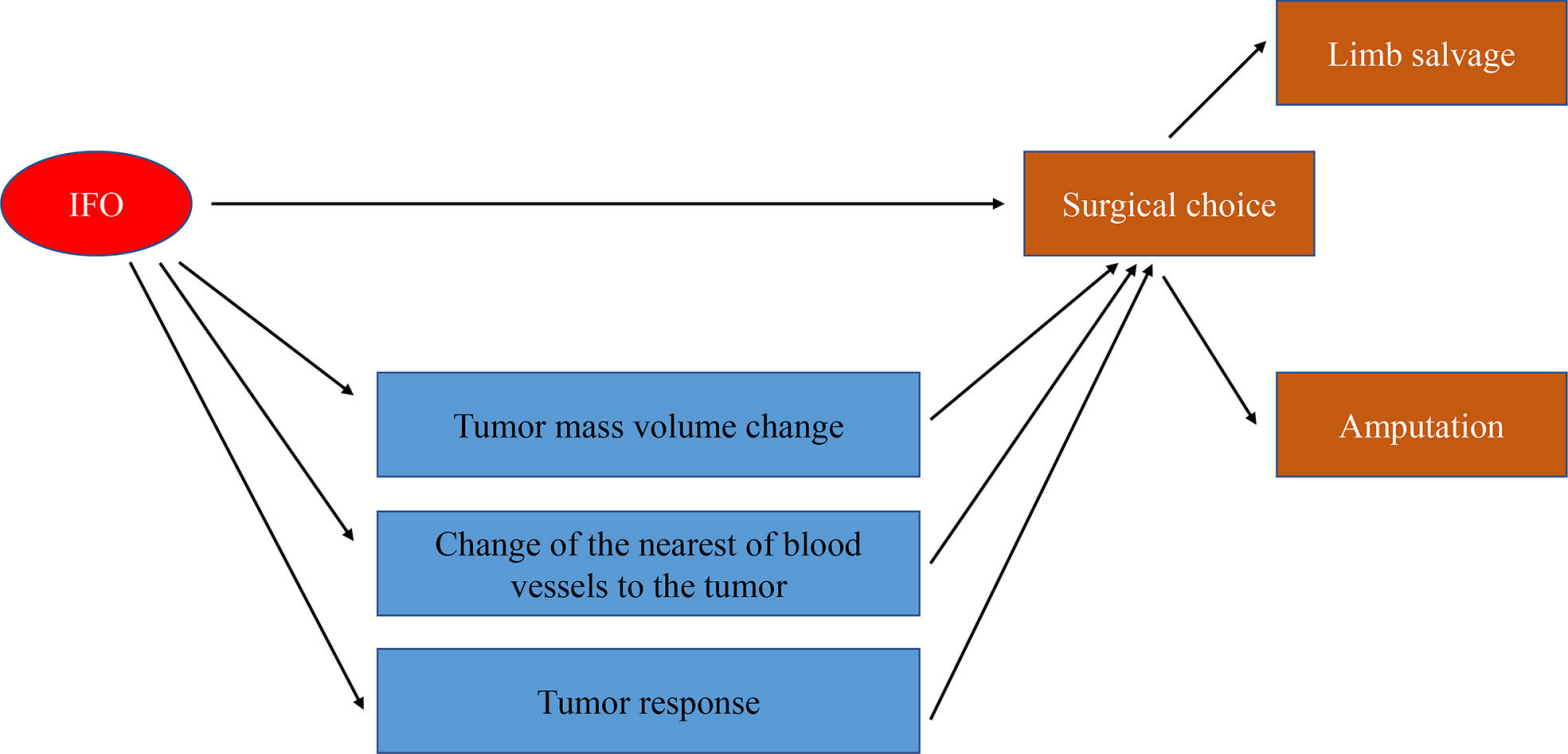
IFO may contribute to limb salvage directly and indirectly.

In summary, we had come to a conclusion that the TMV, NBT, and tumor response were important factors influencing osteosarcoma surgery options. Meanwhile, IFO in neoadjuvant chemotherapy may make contributions to tumor volume shrinkage, increasing the rate of limb salvage and, as a result, providing patients with more opportunities for living quality improvement (**Figure 7**).

## Data availability statement

The original contributions presented in the study are included in the article/**Supplementary Material**. Further inquiries can be directed to the corresponding authors.

## Author contributions

YL wrote the manuscript. YF designed the manuscript. ZZ and ZW revised the manuscript. JS and JY supervised the project. All authors contributed to the article and approved the submitted version.

## Conflict of interest

The authors declare that the research was conducted in the absence of any commercial or financial relationships that could be construed as a potential conflict of interest.

## Publisher’s note

All claims expressed in this article are solely those of the authors and do not necessarily represent those of their affiliated organizations, or those of the publisher, the editors and the reviewers. Any product that may be evaluated in this article, or claim that may be made by its manufacturer, is not guaranteed or endorsed by the publisher.
