# Supplementary material for: Mediating effect assessment of ifosfamide on limb salvage rate in osteosarcoma: A study from a single center in China
Source: Front Oncol. 2022 Nov 3;12:1046199. doi: 10.3389/fonc.2022.1046199 (PMC9669720; doi:10.3389/fonc.2022.1046199)
Supplement: Supplementary file 1 [file Table_1.docx]

Supplementary table1. Comparison information of TMV before and after neoadjuvant chemotherapy of MAP regimen (cm^3^).

| Group | Number | Minimum | Maximum | Median | IQR | Upper quartile | Lower quartile | | Mean | | SD |
| --- | --- | --- | --- | --- | --- | --- | --- | --- | --- | --- | --- |
| Tumor mass before chemotherapy | 46 | 1.69 | 3.30 | 2.90 | 3.01 | 2.47 | 3.12 | 2.92 | | 2.78 | |
| Tumor mass after chemotherapy | 46 | 1.58 | 3.61 | 3.03 | 3.27 | 2.48 | 3.34 | 3.16 | | 3.12 | |
