# Supplementary material for: Mediating effect assessment of ifosfamide on limb salvage rate in osteosarcoma: A study from a single center in China
Source: Front Oncol. 2022 Nov 3;12:1046199. doi: 10.3389/fonc.2022.1046199 (PMC9669720; doi:10.3389/fonc.2022.1046199)
Supplement: Supplementary file 2 [file Table_2.docx]

Supplementary table2. Comparison information of TMV before and after neoadjuvant chemotherapy of MAPI regimen (cm^3^) (*p*=0.004).

| Group | Number | Minimum | Maximum | Median | IQR | Upper quartile | Lower quartile | | Mean | | SD |
| --- | --- | --- | --- | --- | --- | --- | --- | --- | --- | --- | --- |
| Tumor mass before chemotherapy | 239 | 0 | 18.4 | 7 | 4.2 | 5 | 9.2 | 7.084 | | 3.882 | |
| Tumor mass after chemotherapy | 239 | 0 | 28 | 0 | 6 | 0 | 6 | 3.709 | | 5.719 | |
