# Supplementary material for: Mediating effect assessment of ifosfamide on limb salvage rate in osteosarcoma: A study from a single center in China
Source: Front Oncol. 2022 Nov 3;12:1046199. doi: 10.3389/fonc.2022.1046199 (PMC9669720; doi:10.3389/fonc.2022.1046199)
Supplement: Supplementary file 4 [file Table_4.docx]

Supplementary table4. Comparison information of distance of major vessels towards tumor mass margin before and after neoadjuvant chemotherapy of MAPI regimen(cm) ( *p*<0.001).

| Group | Number | Minimum | Maximum | Median | IQR | Upper quartile | Lower quartile | | Mean | | SD |
| --- | --- | --- | --- | --- | --- | --- | --- | --- | --- | --- | --- |
| Tumor mass before chemotherapy | 36 | 0 | 14 | 0 | 6.375 | 0 | 6.375 | 3.306 | | 4.597 | |
| Tumor mass after chemotherapy | 36 | 0 | 26 | 0 | 4.25 | 0 | 4.25 | 3.403 | | 6.647 | |
